# Hematological adjustments of the bony tongue *Arapaima giga*s under influence of amazonian waters

**DOI:** 10.1186/1753-6561-8-S4-P147

**Published:** 2014-10-01

**Authors:** Cleverson Agner Ramos, José Carlos Nunes Raulino, Glauber Cruz de Menezes, Iracimar Batista do Carmo, Elenice Martins Brasil, Elizabeth Gusmão Affonso, Fernandes Marisa Narciso, Daiane Martins

**Affiliations:** 1 Departament of Morphology, Federal University of Amazon, Manaus, Brazil; 2 Department of Aquaculture, National Institute of Amazonian Research (INPA), Manaus, Brazil; 3 Departament of Physiological Sciences, Federal University of São Carlos, São Carlos, Brazil; 4 Post Graduation Program in Biotechnology, Federal University of Amazon (UFAM), Manaus, Brazil

## 

*Arapaima gigas*, populary known as pirarucu is largest freshwater fish in Amazon river, with great, economic and ecological, interest. Despite being an air breather its gill structure is quite close to water breathers, especially in early stages of development [1]. However, in animals above 100g the pillar cell system are embedded into the filament being the gill, an ion regulation active site. The effects of Amazonian rivers waters is well notices in fish [2], mainly by black water (BW), and white water (WW) where the ion fluxes can be meansured [3]. However information on the ionic regulation patters on this species are scarce as well implication by hematological adjustments. Such data could provide inferences on management conditions as well corroborate with what has been suggested by literature, which suggest this species as a panmitic population [4] able to deal with the hydrographic barrier formed by BW in the Amazon basin [5]. The present study aims was to analyzed *A. gigas *hematological parameters when exposed to BW and WW providing suitable hematological data concerning physiological responses in different types of water. Fish were acclimated seven days in three separated ponds containing BW, WW and well water (C), respectively. Control (C) fish were placed in the latter pond. Blood samples were taken from the caudal vessel at the end of acclimation period in order to perform measurement assays on levels of hemoglobin, hematocrit, mean corpuscular volume (MCV), mean corpuscular hemoglobin, mean corpuscular hemoglobin concentration, as well glucose, cholesterol and protein levels. Our findings corroborate the hypothesis stating that BW does interfere on fish adaptation specialy in small fish (~100g). These lack of adaptation should be due the gill morphology of small fish which is close to other water breather fish. However in large fish (~1000g) the findings cleary showed that neither WW or BW can interfere on plasma profile of analysed fish. The compromisse between gas exchange and ion regulation has been demonstrated previously. As hematological adjustment plays a role on such osmorespiratory compromise, the hematological parameters data presented by this study clearly demonstrate that changes in the hematological features play a crucial role in the water to air breathing transition behavior as well in the *A. gigas *gill ontogenetic changes. Despite black water systems being considered a barrier constraining the dispersion of several species; this seems not to be a problem for this species which has kept its ion-regulatory mechanisms even when immerse in black waters.
